# Transcriptional Analysis of *Microcystis aeruginosa* Co-Cultured with Algicidal Bacteria *Brevibacillus laterosporus*

**DOI:** 10.3390/ijerph18168615

**Published:** 2021-08-15

**Authors:** Yulei Zhang, Dong Chen, Ning Zhang, Feng Li, Xiaoxia Luo, Qianru Li, Changling Li, Xianghu Huang

**Affiliations:** 1 Department of Aquaculture, Fisheries College, Guangdong Ocean University, Zhanjiang 524088, China; zhangyl@gdou.edu.cn (Y.Z.); 18348445609@163.com (D.C.); zhangn@gdou.edu.cn (N.Z.); lifeng2318@gdou.edu.cn (F.L.); xialemon@126.com (X.L.); liqianru01@163.com (Q.L.); 2 Shenzhen Research Institute, Guangdong Ocean University, Shenzhen 518108, China

**Keywords:** algicidal bacteria, *Brevibacillus laterosporus*, *Microcystis aeruginosa*, transcriptome, oxidative damage

## Abstract

Harmful algal blooms caused huge ecological damage and economic losses around the world. Controlling algal blooms by algicidal bacteria is expected to be an effective biological control method. The current study investigated the molecular mechanism of harmful cyanobacteria disrupted by algicidal bacteria. *Microcystis aeruginosa* was co-cultured with *Brevibacillus laterosporus* Bl-zj, and RNA-seq based transcriptomic analysis was performed compared to *M. aeruginosa*, which was cultivated separately. A total of 1706 differentially expressed genes were identified, which were mainly involved in carbohydrate metabolism, energy metabolism and amino acid metabolism. In the co-cultured group, the expression of genes mainly enriched in photosynthesis and oxidative phosphorylation were significantly inhibited. However, the expression of the genes related to fatty acid synthesis increased. In addition, the expression of the antioxidant enzymes, such as 2-Cys peroxiredoxin, was increased. These results suggested that *B. laterosporus* could block the electron transport by attacking the PSI system and complex I of *M. aeruginosa*, affecting the energy acquisition and causing oxidative damage. This further led to the lipid peroxidation of the microalgal cell membrane, resulting in algal death. The transcriptional analysis of algicidal bacteria in the interaction process can be combined to explain the algicidal mechanism in the future.

## 1. Introduction

With the increase in water eutrophication, harmful algal blooms (HABs) have frequently occurred worldwide. In HABs, cyanobacteria are the most common group of microalgae, which can produce toxins [1,2,3]. Cyanobacterial blooms can cause serious water environmental problems, such as water oxygen deprivation, odorous substances and toxins, which are harmful to drinking water, aquatic animals, even threatening to human health [4,5,6]. Various methods have been proposed for removing or inhibiting cyanobacterial blooms, which include physical, chemical and biological methods. However, physical and chemical methods are expensive and cause secondary pollution, which is potentially harmful to aquatic ecosystems [7]. In contrast, biological methods are effective strategies to control HABs [8].

Algicidal bacteria are a group of bacteria that can kill or inhibit microalgae. At present, many algicidal bacteria, such as *Bacillus* [9], *Chryseobacterium* [10], *Sulfitobacter* [11], *Hahella* [12] and *Stenotrophomonas* [13], have been previously reported. There are two main mechanisms of algicidal bacteria: (1) direct attack: algicidal bacteria directly invade the microalgal cells; (2) indirect attack: algicidal bacteria inhibit microalgal growth by secreting extracellular active substances to lyse them or by competing with them for nutrients. The first study of algicidal bacteria was reported in *Myxobacter* that inhibited the growth of blue–green algae by secreting some cellulolytic enzymes and digesting the cell wall of the host [14]. Similarly, Furusawa et al. [15] found that *Saprospira* sp. SS98-5 exhibited an algicidal effect on *Chaetoceros ceratosporum,* using its filamentous cells to trap the diatom cells and dissolve their cytoderm, which were in contact with the bacteria. The specific or non-specific extracellular substances secreted by algicidal bacteria during their metabolism can destroy the structure of algal cells and lyse them. Extracellular active substances often act through various mechanisms to dissolve the cyanobacteria, including blocking electron transfer, reducing algal photosynthetic system activity, causing oxidative damage, inhibiting intracellular protein and carbohydrate synthesis and disrupting normal cell metabolism and cellular structure [16,17,18]. Yang et al. found that prodigiosin (PG) from *Hahella* sp. KA22 inhibited *Microcystis aeruginosa* by generating reactive oxygen species (ROS), which induce lipid peroxidation, disrupt the membrane system and obliterate the function of the photosystem [12]. In addition, many proteins of *M. aeruginosa* involved in important metabolic processes in response to PG stress were differentially expressed, which may trigger necrotic-like or apoptotic-like cell death with features similar to those in eukaryotes [12].

The algicidal mechanism has been extensively studied by measuring the physiological and biochemical characteristics of microalgae. A few studies have been conducted on the mechanism of algal-lysing at the molecular and protein levels. RNAseq and microarray analysis were determined to study the interactions between *Roseobacters* and dinoflagellates. The data suggested that at the early mutualistic phase of the symbiosis, polyhydroxyalkanoate (PHA) degradation might be the main carbon and energy source of *Dinoroseobacter shibae*, supplemented in the light by degradation of dimethylsulfoniopropionate (DMSP) and aerobic anoxygenic photosynthesis [19]. Proteomic analysis was applied to investigate the algicidal process of *Trametes versicolor* F21a on *M*. *aeruginosa*, and 30 fungal enzymes with endo- or exoglycosidase activities were significantly up-regulated, suggesting that these enzymes may degrade lipopolysaccharides, peptidoglycans and alginic acid of algal cells [20]. All these studies were analyzed from the algicidal bacteria or fungi, while the analysis of lysed microalgae at a molecular level may provide a new understanding of the algicidal mechanism.

*Brevibacillus laterosporus* is a Gram-positive bacterium that can produce antibacterial molecules and other secondary metabolites that have a wide range of biological activities [21]. In a previous study, the *B. laterosporus* strain Bl-zj was isolated from the intertidal soil of a park in Zhanjiang, which has been proved to have an algae-lytic effect on cyanobacteria, and the potential algicidal factors were screened by genome analysis [22,23].

The research on the mechanism of algicidal bacteria has been mainly conducted using various physiological and biochemical analyses; however, there is a scarcity of reports at the gene level, especially from the lysed microalgae. In this study, the algicidal bacteria *B. laterosporus* Bl-zj and *M. aeruginosa* were co-cultured, and transcriptome analyses were performed to investigate the response of *M. aeruginosa* to algicidal bacteria.

## 2. Materials and Methods

### 2.1. Cyanobacteria and Algicidal Bacterium

*M. aeruginosa* FACHB 905 was purchased from the Freshwater Algae Culture Collection at the Institute of Hydrobiology, Wuhan, China. For inoculum preparation, it was cultured for 7 d to reach the exponential growth phase. It was cultivated in BG11 [24] medium at 28 ± 1 °C under 50 μmol·m^−2^·s^−1^ with a photoperiod of 12:12 h light: dark cycle. The concentration of *M. aeruginosa* was adjusted to 1 × 10^7^ cells/mL for further experiments.

*B. laterosporus* Bl-zj was isolated and preserved by the Laboratory of Algae Resource Development and Culture Environment Ecological Restoration of Guangdong Ocean University. It was cultured using Beef extract–peptone (BP) medium and incubated for 24 h at 28 °C and 150 rpm to reach the logarithmic growth phase. The concentration of *B. laterosporus* was adjusted to 1 × 10^7^ cells/mL for further experiments.

### 2.2. Measurement of the Algicidal Efficiency

Equivalent volumes of microalga and bacterium (10 mL) were mixed, and total culture volume was added to 100 mL with BG11 medium (MB group). The control group was just 10 mL of *M. aeruginosa* added in BG11 medium to the total volume of 100 mL (BG11 group). Each group contained triplicate samples. The experiment lasted 4 days under the same condition as 2.1 when the MB group culture was turned yellow, and the microalgal biomass was at a low proportion.

Spectrophotometer with a 1 cm light path cuvette was used to measure and calculate chlorophyll *a* concentration, according to the methods of Jeffrey and Humphrey [25]. In brief, the cultures of each group were harvested at 0, 1, 2, 3, 4 d, and chlorophyll *a* was extracted with 90% (*v*/*v*) acetone for 24 h in the dark. The absorbance of the supernatant at 630 nm, 647 nm and 664 nm was determined. The chlorophyll *a* concentration was calculated according to the formula: Chl *a* (mg/L) = (11.85 × A_664_ − 1.54 × A_647_ − 0.08 × A_630_) × V_1_/V_2_, where A_630_, A_647_ and A_664_ represent absorbance, V_1_ and V_2_ are the volume of 90% (*v*/*v*) acetone (mL) and cultures (mL), respectively. The removal rate of microalgal cells (R, %) was represented as the algicidal effect and calculated by the formula: R = (C_0_ − C_1_)/C_0_ × 100%, where C_0_ and C_1_ represent the chlorophyll *a* concentration of BG11 group and MB group, respectively.

### 2.3. Transcriptomic Samples Preparation

The *M. aeruginosa* and *B. laterosporus* were co-cultured at a volume ratio of 1:1 (50 mL *M. aeruginosa,* 50 mL *B. laterosporus* and 400 mL BG11 medium). *M. aeruginosa* was separately cultured as control (50 mL *M. aeruginosa* and 450 mL BG11 medium). Samples from the control group (named “CMA”) and experimental group were collected on the second and fourth day (named “MB2” and “MB4”, respectively), as the algicidal effect was obviously on the fourth day. The cocultures were centrifuged at 5000 rpm, 4 °C for 10 min to collect the pellets and then quickly frozen in liquid nitrogen and stored at −80 °C. Each group contained triplicate samples.

### 2.4. Transcriptomic Analysis

Total RNA was extracted using RNAprep Pure Plant Plus Kit (Polysaccharides and Polyphenolics-rich, Tangen, China). Transcriptome sequencing (RNA-seq) was performed by Next-Generation Sequencing on the Illumina HiSeq 4000 platform. To obtain high-quality clean data, the raw reads were filtered by removing adaptor sequences and low-quality reads (Q-value ≤ 20). The clean reads were then mapped onto the *M. aeruginosa* PCC 7806SL complete genome (NCBI reference sequence, CP020771.1) using Bowtie2 program (http://bowtie-bio.sourceforge.net/bowtie2/index.shtml (version 2.4.1, accessed on 28 February 2020)).

The differences in gene expression were analyzed by HTSeq (version0.6.1p2) for the screening of differentially expressed genes (DEGs) and principal components analysis. The read count mapped to each gene was calculated as the original expression of the gene and the FPKM (Fragments Per Kilo bases per Million fragments) was used to normalize the expression levels. The DEGs screening conditions were as follows: log_2_|fold change| > 1, *p*-value < 0.05. DEGs were further annotated to Gene Ontology (GO) database and Kyoto Encyclopedia of Genes and Genomes (KEGG) database.

### 2.5. Quantitative Real-Time PCR Validation

Quantitative PCR (qPCR) was performed to verify the results of mRNA-seq analysis. The cDNA template was synthesized by reverse transcription using a kit (HiScript^R^ III RT SuperMix for qPCR (+gDNA wiper), Vazyme Biotechnology Co., LTD., Nanjing, China). The qPCR was performed using SYBR green (AceQ Universal SYBR qPCR Master Mix, Vazyme Biotechnology Co., LTD., Nanjing, China) on a 7500 Fast Real-time PCR System (Applied Biosystems, Foster, CA, USA). The primers were designed according to the gene sequences from *M. aeruginosa* PCC 7806SL complete genome, with 16S rRNA [26] as the reference gene and synthesized by GENEWIZ (Suzhou, China) (the primers were shown in Supplementary Table S1).

## 3. Results

### 3.1. The Algicidal Efficiency

**Figure 1:**
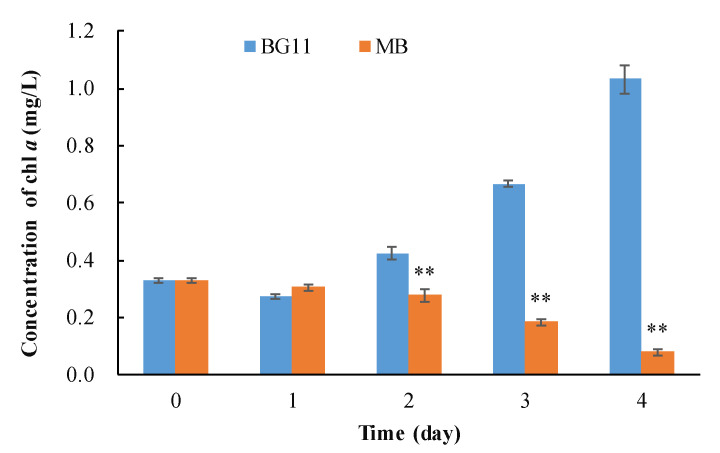
Algicidal effect of *B. laterosporus* on *M. aeruginosa*. (** Represents significant differences to control group at the 0.01 probability level.)

Compared with the control group (BG11), the chlorophyll *a* concentration of *M. aeruginosa* significantly decreased with the addition of B. laterosporus from the second day (Figure 1). The removal rates of *M. aeruginosa* in the MB group were 34.39%, 72.36% and 92.30% on the second, third and fourth days, respectively, showing an increasing algicidal effect over time.

### 3.2. Illumina Sequencing Assembly Data Quality Analysis

Nine cDNA libraries from three groups were sequenced to study the transcriptomes of *M. aeruginosa* under the effect of *B. laterosporus* (Table 1). An average of 9,728,733,733 bp raw data and 64,858,225 raw reads were obtained from each sample. After filtering the adaptors and low-quality sequences from raw data, an average of 7,800,583,533 bp clean data and 52,003,890 clean reads were screened. After mapping to the reference genome, an average of 37,785,780 mapped reads were obtained. All Q20 and Q30 values of the read sequences in the samples exceeded 96.94% and 92.9%, respectively.

**Figure 2:**
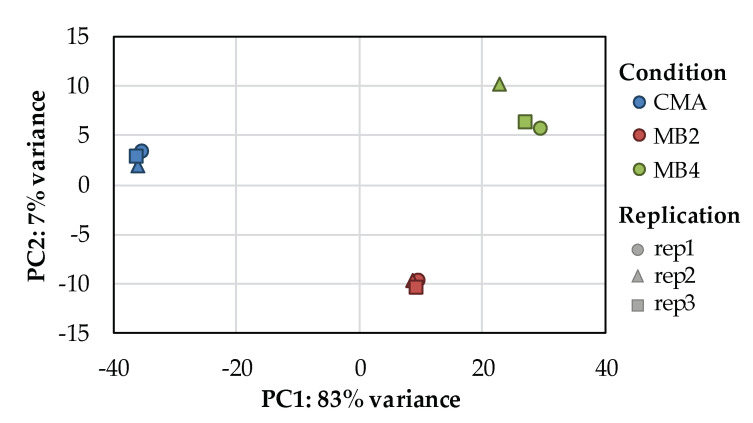
The principal component analysis (PCA) was performed for each sample. (The blue, red and green labels represent the treatment group of CMA, MB2 and MB4, respectively. The circle, triangle and square labels represent replication 1, replication 2 and replication 3 samples in each group, respectively.)

Principal component analysis (PCA) was performed for each sample according to the expression to observe similarities between sample groups (Figure 2). The three replicates of each group (CMA, MB2 and MB4) were gathered with close distance, but different treatment groups separated from others at a long distance, indicating good repeatability data.

### 3.3. Identification of DEGs

**Figure 3:**
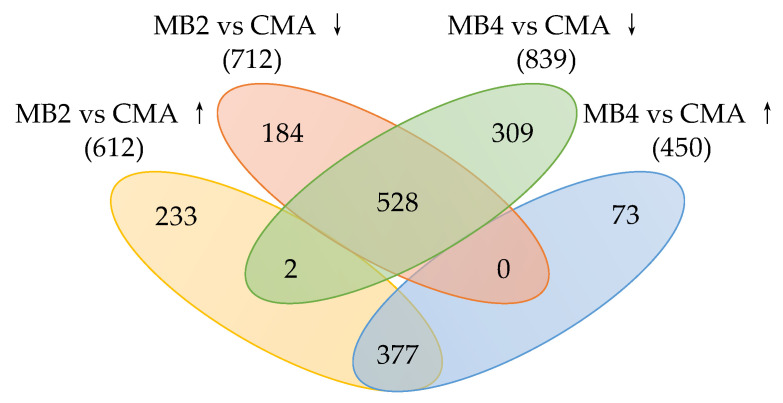
The number of differentially expressed genes (DEGs) at CM2 and CM4 groups compared with CMA group. (“↑” and “↓” represent up-regulated and down-regulated genes, respectively.)

In this study, transcriptomic analysis was used to investigate the impact of algicidal bacteria *B. laterosporus* Bl-zj on *M. aeruginosa* metabolism at the molecular level. Results showed that a total of 1324 DEGs were found in the MB2 group compared to CMA, of which 612 were up-regulated and 712 were down-regulated (Figure 3). There were 1289 DEGs in the MB4 group compared to CMA, of which 450 were up-regulated and 839 were down-regulated. A total of 1706 DEGs were identified; among them, there were 377 up-regulated and 528 down-regulated DEGs both in MB2 vs. CMA comparison and MB4 vs. CMA comparison.

### 3.4. Functional Classification of the DEGs by GO and KEGG Pathway Analysis

The GO and KEGG pathways were used to analyze the DEGs and determine their main biological functions to explore the molecular changes in *M. aeruginosa* under the effect of *B. laterosporus* Bl-zj.

**Figure 4:**
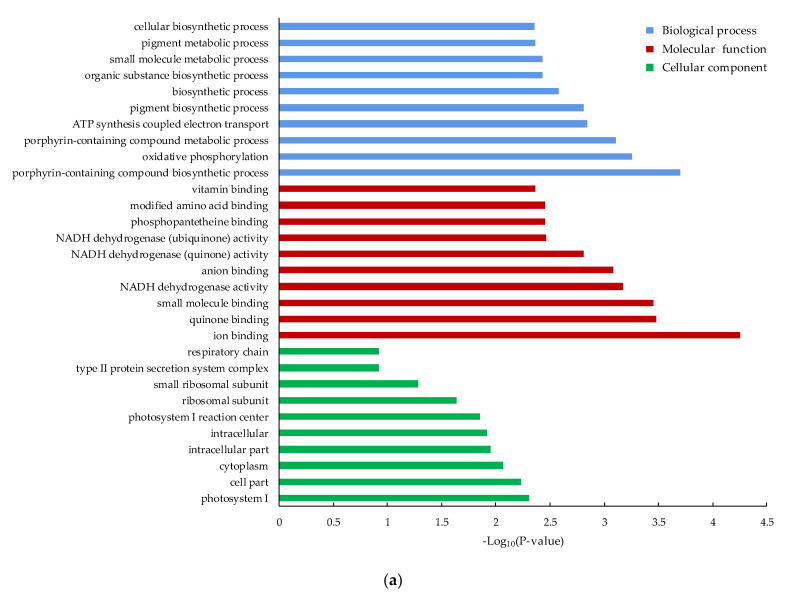

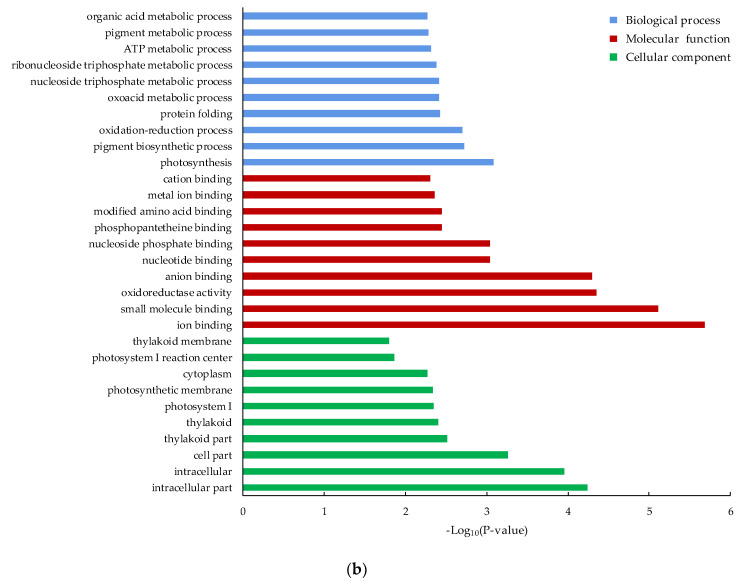
Gene ontology (GO) enrichment analysis of the differently expressed genes at two groups compared with CMA group. (**a**) MB2 vs. CMA comparison; (**b**) MB4 vs. CMA comparison.

The top 10 most significant enriched genes in three GO categories were summarized (Figure 4). In the cellular component category, the GO terms mainly enriched in the MB2 vs. CMA comparison included “photosystem I”, “cell part” and “cytoplasm”. The “intracellular part”, “intracellular” and “cell part” were mainly enriched in the MB4 vs. CMA comparison. Similarly, in the molecular function category, “ion binding”, “quinone binding” and “small molecule binding” were significantly enriched in the MB2 vs. CMA comparison. The “ion binding”, “small molecule binding” and “oxidoreductase activity” were significantly enriched in the MB4 vs. CMA comparison. Further, in the biological process, “prophyrin-containing compound biosynthetic process”, “oxidative phosphorylation” and “prophyrin-containing compound metabolic process” were significantly enriched in the MB2 vs. CMA comparison. The “photosynthesis”, “pigment biosynthetic process” and “oxidation-reduction process” were significantly enriched in the MB4 vs. CMA comparison.

The biological functions associated with DEGs were further analyzed using the KEGG database. The up-regulated and down-regulated genes were involved in carbohydrate metabolism, amino acid metabolism, metabolism of cofactors and vitamins and energy metabolism in MB2 vs. CMA comparison and MB4 vs. CMA comparison (Table 2). In addition, the genes of lipid metabolism and translation were mainly up-regulated and the genes of energy metabolism, replication and repair and membrane transport were mainly down-regulated.

### 3.5. Algicidal-Related Gene and Pathway Analysis

The expression of genes related to photosynthesis, oxidative phosphorylation and fatty acid synthesis in *M. aeruginosa* were significantly changed under the effect of *B. laterosporus* Bl-zj (Table 3). There were nine photosynthesis-related genes that were down-regulated on the second and fourth days, compared to the CMA group, among which seven genes were related to photosystem I (PSI). Nine genes related to oxidative phosphorylation were down-regulated both on the second and fourth days, compared to the control. The expression of ND5 and Ndufs8 were most significantly decreased with log_2_ (fold changes) of 3.38 and 3.43 on the fourth day compared to the control, respectively. In addition, the expression of two genes (FabF and FabG1) involved in fatty acid synthesis increased on the second day compared to the control, while YOXD was increased on the fourth day compared to the control. Similarly, three genes (FabH, FabG2 and FabZ) were up-regulated on the second and fourth days compared to the control.

### 3.6. Quantitative Real-Time PCR Validation

**Figure 5:**
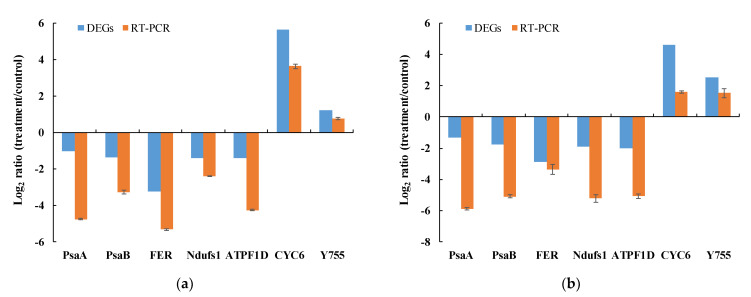
Comparison of the expressions of 7 differently expressed genes determined by Illumina HiSeq 2500 sequencing and qRT-PCR. (**a**) MB2 vs. CMA comparison (**b**) MB4 vs. CMA comparison.

To confirm the RNA-seq results, seven genes were selected for further qRT-PCR analysis. Figure 5a,b shows the expression levels of each gene on the second and fourth days, respectively. Although the fold changes were different, the expression trends of these seven genes were consistent in both RNA-seq and qRT-PCR results.

## 4. Discussion

In the present study, second- and fourth-day transcriptomes of *M. aeruginosa* in co-culture with *B. laterosporus* Bl-zj were obtained and compared using next-generation sequencing technology. We found that several pathways significantly changed, which were probably the critical factors as *B. laterosporus* attacked the cells of *M. aeruginosa*.

Photosynthesis is a critical cellular process that determines the growth of microalgae. In this process, light energy is captured and used to synthesize carbohydrates and produce oxygen while consuming carbon dioxide. Transcriptomic analysis showed that PSI was seriously affected by *B. laterosporus* Bl-zj. The principal subunits of the reaction center (PsaA and PsaB) and other subunits (PsaC, PsaE, PsaD, PsaF and PsaL) were significantly down-regulated on the second day and continuously inhibited on the fourth day (Table 3). PSI is a supercomplex of a reaction center and light-harvesting complexes [27]. PsaA and PsaB are the central core proteins that bonded all cofactors of the electron transport chain except the cluster of ferredoxins and harbor the electron transport chain [28]. PsaC can bind to the terminal electron acceptor and participate in the transfer of electrons to ferredoxin [29]. PsaE was responsible for the dissociation of ferredoxin from the PSI complex and involved in the cyclic electron flow that occurred around the PSI complex [30]. PsaD has been regarded as a key subunit in the assembly, stability and functionality of PSI [31]. PsaF has been found to be related to efficient electron transfer from both plastocyanin and cytochrome c6 to PSI [32]. It has been reported that PsaL was critical for energy transfer from phosphorylated LHCII to the PSI reaction center [33]. In this study, it was observed that Psb28 significantly declined after 2 days compared to the control. Psb28 was the photosystem II reaction center protein, which plays an important role in PSII repair [34]. In addition, plastocyanin and ferredoxin significantly decreased after the second and fourth days compared to the control. Plastocyanin and ferredoxin are small soluble copper protein and iron–sulfur protein, respectively, which are important *PSI* turnover products induced by light energy involved in electron transfer from plastocyanin to ferredoxin [28,35]. These results showed that under the action of *B. laterosporus* Bl-zj photosynthesis, especially the PSI of *M. aeruginosa*, is significantly inhibited. The reduction of photosynthesis-related gene transcripts impeded electron transport, resulting in the loss of equivalent products necessary for the process of carbon assimilation.

Oxidative phosphorylation is the main energy source of aerobic cells and the main pathway of ATP production [36]. It is an enzymatic process in which ATP is formed when electrons are transferred from substrate to oxygen and ADP is phosphorylated. Transcriptomic analysis showed that the genes related to oxidative phosphorylation significantly decreased after the second and fourth days, relative to the control (Table 3). NADH: ubiquinone oxidoreductase (Complex I) is the first and most complex enzyme required in this process. It connected NADH and ubiquinone electron transfer to proton transmembrane transport, which helped to generate proton power, which was necessary for ATP synthesis. Further, ND1, ND2, ND4, ND5, Ndufs1, Ndufs8 were the core subunits of Complex I [37]. In *Chlamydomonas*, the loss of ND1 or ND5 subunit prevented the assembly of whole mitochondrial Complex I, whereas the loss of ND4 led to the formation of a subcomplex of 650 kDa present in a reduced amount [38,39]. In addition, COX10 related to cytochrome c oxidase (Complex IV) and ATPF_0_B, ATPF_1_D related ATP synthase significantly decreased after the second and fourth days compared to the control group. Complex IV catalyzes the last step of the mitochondrial electron transfer chain and is considered one of the main regulatory sites of oxidative phosphorylation [40]. COX10 participates in the biosynthesis of heme a, a key cofactor of cytochrome c oxidase [41]. ATP synthase can synthesize cellular ATP from ADP and inorganic phosphate. It is comprised of two parts, an ATP-driven motor F_1_ and a proton-driven F_0_ motor [42].

The down-regulation of oxidative phosphorylation expression led to a reduction in the transmembrane gradient and ATP synthesis, suggesting that *B. laterosporus* Bl-zj decreased the amount of cellular ATP in *M. aeruginosa* cells. Moreover, the absolute value of log_2_ (fold changes) of MB4 vs. CMA comparison were almost larger than that of MB2 vs. CMA comparison. This indicated that oxidative phosphorylation of *M. aeruginosa* was continuously inhibited. Further, the ATP synthesis decreased, and the cyanobacteria cells could not obtain enough energy to survive. In addition, the electron transport chain and oxidative phosphorylation are critical cellular processes that sustain life, and the absence of either can affect cellular respiration and even lead to cell death [43]. The decrease of electron transport chain and oxidative phosphorylation genes expression could inhibit the algal growth and even cause death.

The response and adaptability of microalgae to environmental changes were closely related to numerous changes in the composition of lipids in cells and the ability to synthesize a series of special lipids [44]. Under stress, some algal cells cannot reproduce but accumulate lipids instead [45,46]. Many deep-sea bacteria adapted to the deep-sea environment by synthesizing more unsaturated lipids [47]. In this study, the expression of fatty acid biosynthesis-related genes FabH and FabZ were up-regulated in both MB2 and MB4 groups compared to control, while FabF was up-regulated only in MB2 vs. CMA comparison (Table 3). FabF catalyzed the elongation of fatty acyl chains and NADPH-specific reduction of long-chain β-ketoacyl derivatives [48]. FabH was considered to catalyze the first elongation reaction (Claisen condensation) of type II fatty acid synthesis, resulting in the production of short-chain fatty acid primers [49]. FabZ showed ubiquitous distribution in type II fatty acid synthase, and the dehydratase efficiently catalyzed the dehydration of short and long-chain saturated and unsaturated beta-hydroxyacyl-ACPs [50]. The up-regulated expression of fatty acid synthesis genes revealed that *M. aeruginosa* was under stress. When *M. aeruginosa* was under the stress of pyrogallol, a compound with strong inhibition on *M. aeruginosa*, the expression of FabZ was also increased [51]. Moreover, the expression of gene Y755 was up-regulated. 2-Cys peroxiredoxin was a subclass of peroxiredoxin proteins (Prx-s), and Prx-s were a ubiquitous family of antioxidant enzymes and important components of the cellular antioxidant defense system as well as redox homeostasis [52]. These suggested that membranes may undergo oxidative damage. In order to maintain the normal function of membranes, more unsaturated fatty acids need to be integrated into membranes.

## 5. Conclusions

In the present study, we provide the molecular responses of *M. aeruginosa* to *B. laterosporus* treatment. The *B. laterosporus* treatment led to a suppression of photosynthesis and oxidative phosphorylation of *M. aeruginosa.* We also found that the expression of the genes related to fatty acid synthesis increased. In addition, the expression of the antioxidant enzymes, such as 2-Cys peroxiredoxin, was increased. The inhibition of photosynthesis and oxidative phosphorylation of *M. aeruginosa* could block electron transport and affect energy acquisition. The increase in fatty acid synthesis-related genes and 2-Cys peroxiredoxin indicated that cyanobacteria exhibited oxidative damage, causing algal cell membrane lipid peroxidation. These changes inhibited cyanobacteria growth, leading to algae death.

## Figures and Tables

**Table 1:** Summary of transcriptome sequencing data of *M. aeruginosa* under *B. laterosporus*.

Sample	Raw Data (bp)	Raw Reads No.	Clean Data (bp)	Clean Reads No.	Mapped Reads No.	Q20 (%)	Q30 (%)
CMA1	9,468,720,000	63,124,800	8,089,035,900	53,926,906	52,199,272	97.86	94.09
CMA2	9,206,526,300	61,376,842	7,486,911,900	49,912,746	48,062,591	97.93	94.40
CMA3	8,543,553,900	56,957,026	7,371,049,200	49,140,328	47,594,707	98.05	94.50
MB2_1	11,260,710,900	75,071,406	9,110,216,700	60,734,778	43,388,415	97.73	94.22
MB2_2	9,961,599,300	66,410,662	7,954,650,900	53,031,006	39,017,497	97.62	93.88
MB2_3	9,498,113,100	63,320,754	7,660,962,000	51,073,080	36,364,637	97.65	93.90
MB4_1	10,501,108,500	70,007,390	8,108,475,900	54,056,506	37,308,387	97.72	94.15
MB4_2	9,475,501,500	63,170,010	7,402,896,600	49,352,644	4,777,919	97.29	93.41
MB4_3	9,642,770,100	64,285,134	7,021,052,700	46,807,018	31,358,598	96.94	92.90
Average	9,728,733,733	64,858,225	7,800,583,533	52,003,890	37,785,780	97.64	93.94

**Table 2:** Distribution of up- and down-regulated genes in KEGG pathways.

KEGG Pathways (Level 2)	MB2 vs. CMA		MB4 vs. CMA	
	Up	Down	Up	Down
Amino acid metabolism	42	25	39	38
Carbohydrate metabolism	59	40	52	60
Energy metabolism	31	44	27	52
Metabolism of cofactors and vitamins	33	15	28	23
Lipid metabolism	15	6	16	11
Translation	16	3	14	2
Metabolism of other amino acids	9	4	9	4
Biosynthesis of other secondary metabolites	7	4	9	5
Nucleotide metabolism	12	11	7	15
Metabolism of terpenoids and polyketides	8	7	7	4
Replication and repair	6	10	5	14
Membrane transport	4	8	2	9
Xenobiotics biodegradation and metabolism	5	2	3	3
Transcription	2	1	2	1
Signal transduction	1	4	1	3
Immune system	0	2	0	2
Glycan biosynthesis and metabolism	2	2	1	4
Folding, sorting and degradation	8	3	5	8
Cell growth and death	1	1	1	1
Cellular community—prokaryotes	1	7	1	8
Environmental adaptation	-	-	0	1

**Table 3:** Potential candidate genes related to the response to *B. laterosporus* Bl-zj treatment (FC = fold change).

Gene ID	Symbol	MB2 vs. CMA		MB4 vs. CMA		Annotation
		log_2_FC	*p*-Value	log_2_FC	*p*-Value	
**Photosynthesis**						
RS02885	PsaA	−1.02	1.66 × 10^−3^	−1.29	1.35 × 10^−3^	photosystem I core protein psaA
RS02890	PsaB	−1.34	2.02 × 10^−5^	−1.73	8.05 × 10^−6^	photosystem I core protein psaB
RS22460	PsaC	−2.5	1.13 × 10^−15^	−3.97	3.25 × 10^−19^	photosystem I subunit VII
RS24715	PsaD	−2.93	2.42 × 10^−23^	−4.04	6.13 × 10^−23^	photosystem I reaction center subunit II
RS04855	PsaE	−1.66	6.27 × 10^−13^	−1.97	6.76 × 10^−7^	photosystem I reaction center subunit IV
RS02775	PsaF	−1.61	3.01 × 10^−13^	−1.96	8.76 × 10^−8^	photosystem I subunit III
RS25020	PsaL	−3.61	1.36 × 10^−53^	−3.71	3.78 × 10^−19^	photosystem I reaction center subunit XI
RS13445	PC	−4.00	8.78 × 10^−65^	−3.40	3.01 × 10^−22^	plastocyanin
RS12310	FER	−3.24	4.87 × 10^−29^	−2.88	8.76 × 10^−13^	ferredoxin
RS15355	Psb28	−1.12	2.85 × 10^−2^	-	-	photosystem II reaction center protein Psb28
RS02780	PsaJ	−1.61	4.15 × 10^−6^	-	-	photosystem I reaction center subunit IX
RS15435	UCRI	-	-	−1.6	9.44 × 10^−5^	cytochrome b6-f complex iron-sulfur subunit 1
RS16845	ISIA	3.94	1.97 × 10^−61^	3.36	6.36 × 10^−21^	iron stress-induced chlorophyll-binding protein
RS13450	CYC6	5.63	2.69 × 10^−17^	4.63	2.31 × 10^−4^	cytochrome c6
RS12320	PsbU	-	-	1.17	2.53 × 10^−3^	photosystem II 12 kDa extrinsic protein
RS16660	PsbJ	1.08	1.00 × 10^−4^	-	-	photosystem II reaction center protein J
**Oxidative phosphorylation**						
RS07425	ND1	−1.38	7.03 × 10^−9^	−1.77	2.21 × 10^−4^	NAD(P)H-quinone oxidoreductase subunit 1
RS05160	ND2	−1.09	3.98 × 10^−7^	−1.72	5.03 × 10^−6^	NAD(P)H-quinone oxidoreductase subunit 2
RS13045	ND4	−2.57	3.58 × 10^−10^	−2.61	1.02 × 10^−5^	proton-translocating NADH-quinone oxidoreductase, chain M family protein
RS13470	ND5	−2.38	1.77 × 10^−16^	−3.38	1.70 × 10^−7^	NAD(P)H dehydrogenase, subunit NdhF3 family protein
RS08000	Ndufs1	−1.39	4.18 × 10^−6^	−1.89	2.86 × 10^−6^	2Fe-2S iron-sulfur cluster binding domain protein
RS07420	Ndufs8	−2.64	3.79 × 10^−14^	−3.43	6.95 × 10^−15^	NADH-plastoquinone oxidoreductase, I subunit
RS23775	COX10	−1.35	6.50 × 10^−9^	−2.27	2.37 × 10^−11^	protoheme IX farnesyltransferase
RS09740	ATPF1D	−1.39	1.65 × 10^−4^	−2.01	1.96 × 10^−3^	ATP synthase F1, delta subunit
RS09735	ATPF0B	−1.57	1.47 × 10^−6^	−2.33	1.05 × 10^−7^	ATP synthase F0, B subunit
RS24375	ND3	−2.33	1.35 × 10^−3^	-	-	NADH dehydrogenase subunit A
RS05535	Ndufv2	−1.12	1.16 × 10^−6^	-	-	respiratory-chain NADH dehydrogenase 24 kDa subunit
RS23755	COX3	-	-	−1.64	9.83 × 10^−4^	cytochrome c oxidase subunit III
RS09750	ATPG	-	-	−1.5	1.37 × 10^−3^	ATP synthase gamma chain
RS12325	NADB	1.5	3.14 × 10^−7^	-	-	L-aspartate oxidase
RS23760	COX1	1.37	7.16 × 10^−9^	-	-	cytochrome c oxidase, subunit I
RS23765	COX2	1.1	2.64 × 10^−7^	-	-	cytochrome c oxidase, subunit II
RS24380	Ndufs7	-	-	1.45	9.08 × 10^−5^	NAD(P)H-quinone oxidoreductase subunit K 1
RS13440	DHSA	-	-	1.53	9.29 × 10^−5^	succinate dehydrogenase/fumarate reductase, flavoprotein subunit
**Fatty acid biosynthesis**						
RS04585	FabH	1.88	3.38 × 10^−11^	1.97	1.56 × 10^−2^	3-oxoacyl-[acyl-carrier-protein] synthase III
RS18070	FabG2	1.31	2.24 × 10^−9^	1.33	1.12 × 10^−2^	PHA-specific acetoacetyl-CoA reductase
RS08035	FabZ	2.42	4.01 × 10^−13^	2.51	6.76 × 10^−5^	beta-hydroxyacyl-(acyl-carrier-protein) dehydratase
RS22350	FabF	1.32	6.66 × 10^−4^	-	-	beta-ketoacyl synthase, C-terminal domain protein
RS12750	FabG1	1.16	9.88 × 10^−5^	-	-	3-oxoacyl-[acyl-carrier-protein] reductase
RS05845	YOXD	-	-	2.26	3.16 × 10^−9^	short chain dehydrogenase family protein
RS23000RS09855	AAE16	−2.73	8.26 × 10^−10^	−4.54	3.16 × 10^−11^	AMP-binding enzyme family protein
	FabI	-	-	−1.79	4.63 × 10^−3^	enoyl-[acyl-carrier-protein] reductase [NADH]
**Antioxidase**						
RS02415	Y755	1.21	2.98 × 10^−8^	2.52	4.86 × 10^−12^	2-Cys peroxiredoxin BAS1

## Supplementary Materials

The following are available online at https://www.mdpi.com/article/10.3390/ijerph18168615/s1, Table S1: PCR primer pairs used in this study.
