# Supplementary material for: Transcriptional Analysis of Microcystis aeruginosa Co-Cultured with Algicidal Bacteria Brevibacillus laterosporus
Source: Int J Environ Res Public Health. 2021 Aug 15;18(16):8615. doi: 10.3390/ijerph18168615 (PMC8394347; doi:10.3390/ijerph18168615)
Supplement: Supplementary file 1 [file ijerph-18-08615-s001.zip › ijerph-1282221-SI.pdf]

Supplementary table 1. PCR primer pairs used in this study.

Gene id	Gene name	Forward Sequence (5′-3′)	Reverse Sequence (5′-3′)
RS02885	psaA	ACACTTGGATCGGTGGCTTC	GCATGGTGTCGTTGTGAATGT
RS02890	psaB	ATCGCTTACACCGCCTATCC	AACCACGAGCGTCTAAAGCA
RS12310	FER	GAAGCAGGACTGGACTTACCT	CAGAGGTAGGATAGGCGACG
RS08000	Ndufs1	GAAGTGGCGGAAGGAATGGT	TCGCTTGGGAAAACGGTAGG
RS09740	atpF1D	GGACCAGTGCCAATCCCTATT	CCGGTCAGGGTTTTCACTTTG
RS13450	CYC6	GTATGGCTCGTCCCGCTTTA	GCATCGCACCGTTACCTTTG
RS02415	Y755	GGCCTATCCTTTGGTGTCGG	TTCAGAGTGCGGAGGGTTTC
	16S rRNA	CTAAAGGCGGTGGAAACTGG	CGGCTAGGACTACAGGGGTATCT
